# Towards experimental quantum-field tomography with ultracold atoms

**DOI:** 10.1038/ncomms8663

**Published:** 2015-07-03

**Authors:** A. Steffens, M. Friesdorf, T. Langen, B. Rauer, T. Schweigler, R. Hübener, J. Schmiedmayer, C.A. Riofrío, J. Eisert

**Affiliations:** 1 Dahlem Center for Complex Quantum Systems, Freie Universität Berlin, Berlin 14195, Germany; 2 Vienna Center for Quantum Science and Technology, Atominstitut, TU Wien, Stadionallee 2, Vienna 1020, Austria

## Abstract

The experimental realization of large-scale many-body systems in atomic-optical architectures has seen immense progress in recent years, rendering full tomography tools for state identification inefficient, especially for continuous systems. To work with these emerging physical platforms, new technologies for state identification are required. Here we present first steps towards efficient experimental quantum-field tomography. Our procedure is based on the continuous analogues of matrix-product states, ubiquitous in condensed-matter theory. These states naturally incorporate the locality present in realistic physical settings and are thus prime candidates for describing the physics of locally interacting quantum fields. To experimentally demonstrate the power of our procedure, we quench a one-dimensional Bose gas by a transversal split and use our method for a partial quantum-field reconstruction of the far-from-equilibrium states of this system. We expect our technique to play an important role in future studies of continuous quantum many-body systems.

## Abstract (web summary)

Full tomography of the quantum state of a many-body system becomes harder as more and more atoms are included. Here the authors borrow a concept from condensed-matter physics, continuous matrix-product states, and present an efficient approach for experimental quantum-field tomography.

## Graphical abstract

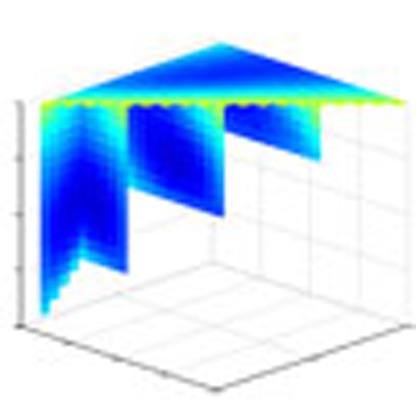

Complex quantum systems with many degrees of freedom can now be controlled with unprecedented precision, giving rise to applications in quantum metrology^1^, quantum information^1^,^2^ and quantum simulation^3^,^4^. This holds true specifically for architectures based on trapped ions^5^ and ultracold atoms^3^,^6^,^7^,^8^, where large system sizes can now routinely be realized, while still maintaining control down to the level of single constituents. In the light of this development, the mindset has shifted when it comes to the assessment and verification of preparations of quantum states. Traditionally, experiments are being used as a vessel to test the validity of theoretical models by comparing their predictions to specific experimental output. With quantum experiments of many degrees of freedom becoming significantly more accurate, an attitude of ‘quantum engineering' and quantum simulation is taking over. Compared with the traditional mindset, one does not compare the experimental data to predictions from theoretical models, but rather uses the full capabilities of the experimental setup as an investigative tool for the physical situation at hand. Triggered by this development and driven by the goal to maximize the information extracted from the experiment, the standards in quantum system identification have substantially risen. Quantum-state tomography^9^,^10^,^11^ fulfils this need for precise and model-independent quantum-state identification. It asks the question: given data, what is the unknown quantum state compatible with those data? Maybe unsurprisingly, the interest in the field of quantum system identification and quantum-state tomography has exploded in recent years^10^,^11^,^12^,^13^.

For many degrees of freedom, unqualified quantum state tomography must be inefficient in the system size, as exponentially many numbers need to be specified. This problem has given way to the insight that practically only the states found in experiments need to be reconstructed, which form only a small subset of the full Hilbert space^14^,^15^. Accordingly, more efficient tomography tools^9^ have been developed, ranging from quantum compressed sensing^10^ (for states of approximately low rank), over permutation-invariant tomography, to matrix-product state tomography^11^,^12^,^13^,^16^. These approaches are based on using the right ‘data set' having the appropriate ‘sparsity structure' to capture quantum many-body systems. For discrete systems, matrix-product states efficiently capture the low-energy behaviour of locally interacting models and a large body of literature in the condensed-matter context backs up this intuition of the ‘physical corner of Hilbert space'^14^,^15^,^17^.

In this work, we consider continuous systems, in which the tomographic problem is aggravated due to the fact that, in principle, infinitely many degrees of freedom need to be reconstructed. On the basis of the notion of sparsity, we present a novel quantum-field tomography procedure relying on the class of continuous matrix-product states (cMPS)^18^,^19^. This approach will allow us to give evidence that the state encountered in the laboratory is well approximated by a representative of this class.

## Results

### Quantum-field tomography

We apply our procedure to non-equilibrium experiments of a continuous quantum gas of one species of bosonic particles whose correlation behaviour can be captured by translation invariant states of the form


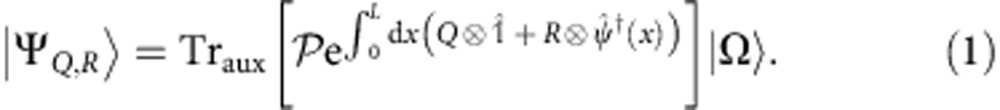


Here 
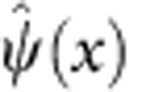
, *x*∈[0, *L*] are the canonical bosonic field operators, 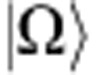 is the vacuum state vector, *Q*, *R*∈C^*d* × *d*^ are matrices acting on an auxiliary d-dimensional space and completely parametrize the state. *L* is the length of the closed physical system, 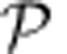 denotes the path ordering operator and Tr_aux_ traces out the auxiliary space. The bond dimension *d* takes the same role as the bond dimension for matrix-product states: Low entanglement states are expected to be well approximated by cMPS of low bond dimension; in turn, for suitably large *d*, every quantum-field state can be approximated.

We employ our reconstruction procedure to perform quantum state tomography for a one-dimensional (1D) system of ultracold Bose gases, an architecture that provides one of the prime setups for exploring the physics of interacting quantum fields^6^,^20^,^21^. The experiment consists of a large 1D quasi-condensate that is trapped using an atom chip^22^. To bring the system out of equilibrium, a split transversal to the condensate direction is performed. The subsequent out-of-equilibrium dynamics after the quench leads to apparent equilibration, prethermalization and thermalization^6^,^23^,^24^. In the middle of the trap, the system can be well approximated by two parallel quantum fields that are homogeneous and translationally invariant.

The experiment proceeds by performing a joint time-of-flight measurement of the two quasi-condensates. Since the experimentally measured images are single-shot measurements, repeating the experiment many times with identical initial conditions allows to extract the phase difference 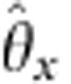 of the two quasi-condensates at different longitudinal position *x* and construct higher order correlation functions^6^,^25^. The phase correlation functions are defined as


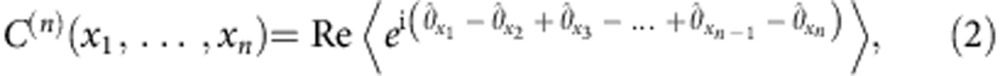


where 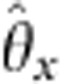 are the measured phase differences and the angular brackets denote the ensemble average (Methods section).

To capture these correlation function in terms of a cMPS, we use a description in terms of effective field operators for the phase difference


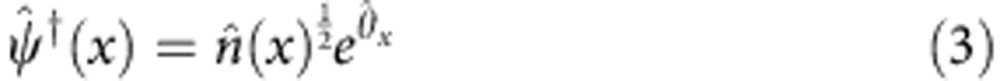


where 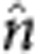 are density operators. As no density information could be obtained from the experiment in its current form, the expectation value of these operators remains unknown and our work is a partial reconstruction of the state. However, the obtained cMPS contains its full phase correlation behaviour.

Using this description, we can write an *n*-point phase correlation functions as


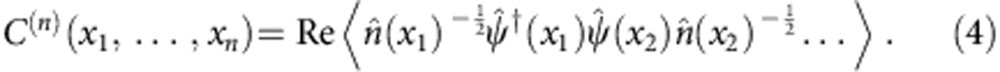


Since it is sufficient for performing the tomography procedure, we will use the correlation information of the normal ordered subset with *x*_1_≤*x*_2_≤⋯≤*x*_*n*_ of the even-order correlation functions. In the cMPS language, assuming translation invariance and the thermodynamic limit, this can be reformulated as


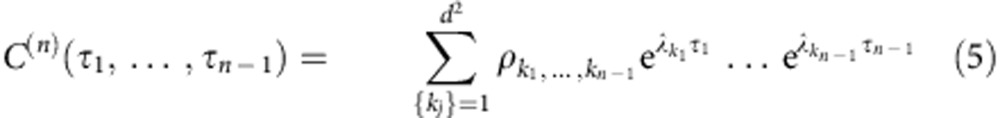


with *τ*_*k*_=*x*_*k*+1_−*x*_*k*_,


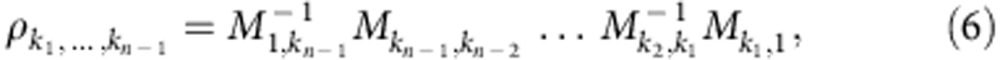


*λ*_*k*_ being the eigenvalues of the transfer matrix *T*, and *M* being 
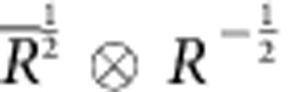
 in the diagonal basis of *T* (Methods section)^16^. The reconstruction proceeds by first extracting the eigenvalues *λ*_*k*_ from the two-point correlation function and in a second step, determining a compatible *M* matrix^26^ from the four-point correlators.

### Data analysis

We find that a cMPS with *d*=2, corresponding to four reconstructed poles and a 4 × 4 matrix *M*, matches the data. This indicates that the correlation function has a simple structure as one would expect from such local physical interactions (specifically based on previously explored descriptions in terms of a Luttinger liquid theory^6^). More importantly, no previously known theoretical description of the physical situation at hand is needed since the cMPS ansatz can be applied to any locally interacting quantum field. To estimate the performance of the reconstruction of the four-point correlation function, we use the mean relative deviation (Methods section), and find a small error of 1.4%, which is of the same magnitude as the experimental errors^6^.

**Figure 1:**
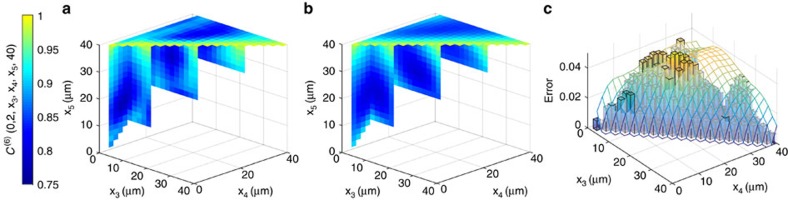
Projections of the measured and predicted six-point correlation function. We show projections of the relevant sections of the (**a**) experimental and (**b**) predicted six-point function for a hold time after the quench of *t*=3 ms. This image shows the volumetric elements of certain projections of the high-dimensional six-point correlation function array and demonstrates a great overall agreement between experimental data and the predicted correlation data. In **c**, the absolute difference between the experimental and the predicted data points for the projection *C*^(4)^(0, 2, *x*_3_, *x*_4_) is shown as a bar plot, the statistical uncertainties of the data as a transparent mesh. More quantitatively, as a figure of merit for measuring the performance of the reconstruction, we use the mean relative deviation over all indices belonging to the relevant simplex of the data with *x*_1_≤ *x*_2_ ≤···≤ *x*_6_ (Methods section) and find a mean error of 2.5% and a maximum relative deviation of 9.1%.

Approximating a correlation function can be done in many ways and it is, *a priori*, not clear that one has truly gained knowledge about the state. The advantage of the cMPS ansatz is that the approximation performed is sufficient to fully reconstruct the phase correlation behaviour of the cMPS. We build trust in the reconstructed state by using it to predict higher order correlation functions, which in turn can be experimentally checked. This provides an excellent benchmark for our procedure and allows us to estimate the quality of our guess for the unknown experimental state. Specifically, we obtain an error of 3.2% for the six-point function (Fig. 1), estimated with bootstrapping techniques. This shows that the reconstruction of the full correlation behaviour of the state was successful, providing a proof-of-principle application for efficient state tomography of interacting many-body quantum fields.

**Figure 2:**
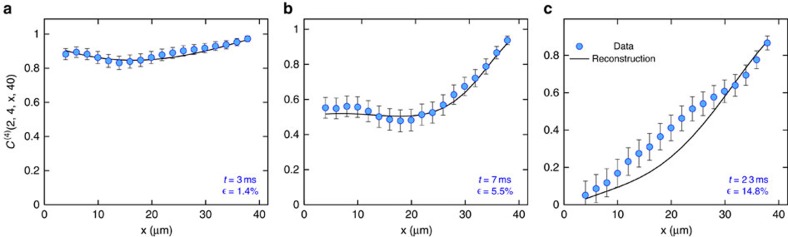
Projections of the four-point correlation function. We show projections of the four-point correlators for a hold time of (**a**) 3 ms, (**b**) 7 ms and (**c**) 23 ms. The quality of the cMPS ansatz decreases substantially with the hold time, with a mean relative deviation 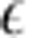 of the full four-point correlator as indicated in the figures. This increase of the deviation with hold time could be seen as an indicator for the non-equilibrium processes in the system (see main text), but is presumably also related to the increase in s.e. in the experiment, as indicated by the error bars (Methods section).

We have performed our reconstruction of the six-point correlator for different hold times after the quench and observe that the fit quality drops substantially with increasing time with mean relative deviations of 3.2%, 10.7% and 34.1% for times *t*=3, 7 and 23 ms, respectively (Fig. 2). There are several possible explanations for this decrease in reconstruction quality. While quantum-field tomography necessarily has to rely on a finite-dimensional ‘data set', it is clear that not all situations can be captured equally well by the approach proposed here. This method applies to states of low entanglement, a situation expected to be present for ground states or states in non-equilibrium following quenches for short times. It will surely be difficult to capture highly entangled or thermal states, which are expected to have a high description complexity, with these tools^26^.

## Discussion

The physics of sudden quenches in discrete settings is usually connected to a linear entanglement growth with time^15^,^23^,^27^, while for each time satisfying an area law in space^15^. Note that while the continuous physical system at hand can be well captured with a free Tomonaga–Luttinger liquid model^28^,^29^, the states of the system can still be strongly entangled, in the sense that entanglement entropies across any real-space cut of the system are, in principle, arbitrarily large. It is precisely this spatial entanglement that will surely influence the quality of tensor network descriptions of the state and that is a key factor for the quality of any cMPS reconstruction^26^. Since our cMPS reconstruction with *d*=2 is only well-suited for states with low entanglement, a similar entanglement buildup for the performed sudden quench of quantum fields would be a natural explanation. Indeed, such light cone dynamics for the correlations of these systems^6^,^30^,^31^ have recently been made explicit experimentally. Such entanglement growth could conceptually be unveiled by investigating how the fit quality changes when the bond dimension is increased. Given the structure of the data set (analysis contained in the Methods section) and the increase of experimental errors with hold time, the exploration of this observation lies outside the scope of this work, but is surely an interesting topic for the near future.

Experimental imperfections or the remaining actual temperature could be other sources for the decrease in fit quality with hold time, as they lead to a mixed state, thus impeding our description in terms of pure states. Previous studies, however, successfully described the system in terms of a pure state Luttinger liquid, even for long evolution times^31^. Moreover, the experimental data was taken in the middle of the trap, where, initially, the assumption of translational invariance holds up to excellent accuracy. For long hold times after the quench, however, regions outside of the center of the trap will have an influence on the behaviour of the system in the middle^6^, thus making the data less translational invariant (Methods section).

The work presented here is surely a first step in the direction of a larger programme, advocating a paradigm change in the evaluation of experimental data from atomic-optical architectures. Instead of comparing predictions of an assumed theoretical model with data, one puts the data into the focus of attention and attempts a reconstruction in the mindset of quantum tomography. This, in particular, seem an important development in the context of quantum simulators, which have the potential to address questions on interacting quantum systems that are inaccessible with classical means. While partial information of the results of a quantum simulator can easily be accessed, a full read-out necessarily corresponds to performing quantum tomography where feasible tools are still lacking. The present work offers a step forward and presents a novel tool to obtain and build trust in the complete results of a quantum simulation without having to include any information of the underlying Hamiltonian of the system.

## Methods

### Experiment

A single specimen of an ultracold gas of ^87^Rb atoms is prepared using evaporative cooling on an atom chip. The final temperature and the chemical potential of the gas are both well below the first radially excited state of the trapping potential, implementing a 1D bosonic system that is well approximated by the Lieb–Liniger model. The systems contain several thousand atoms and spread over sizes as large as 100 μm. A sudden global quench is realised by transversally splitting the gas into two mutually coherent halves^32^, leading to an out-of-equilibrium, approximately pure state. The setup in principle allows for different splitting procedures, in particular an experimental scheme to test the Unruh effect with a specially modelled split has recently been proposed^33^. Subsequently, this non-equilibrium system is let to evolve in the trap for a variable hold time. Its dynamical states are probed using matter wave interferometry in time-of-flight, which enables the direct measurement of the local relative phase *θ*_*x*_. Since the experimentally measured images are single-shot measurements, repeating the experiment many times with identical initial conditions allows to measure not only the mean of the correlations, but also higher order correlation functions are accessible^6^. The corresponding correlation functions are constructed by averaging over ∼150 experimental realizations.

We are restricted to even-order correlation functions in the experiment. The reason for this is the fact that many experimental realizations are needed to construct the correlation functions. Each of these experimental realizations provides us with a measurement of the relative phase *θ*_*x*_=*φ*(*x*)+*ϕ*. Here *φ* is the actual fluctuating phase that contains the interesting many-body physics and *ϕ* is a small global phase diffusion that is random in every experimental realization^32^. This global phase diffusion results from small shot-to-shot fluctuations in the electrical currents that create the trapping potential. These cause small random imbalances of the double well, leading to random and unknown values for *ϕ*. For the even-order correlation functions only differences between the *θ* at different positions need to be evaluated. Consequently, the global shifts ϕ cancel automatically. However, for odd-order correlation functions contributions ∼*e*^*iϕ*^ remain. Hence, the measured result does not only contain the pure dynamics, but is significantly perturbed by the unknown fluctuations of *ϕ*.

### Reconstruction procedure

To make the correlation function in equation 2 directly accessible to our reconstruction procedure, we write it in terms of field operators 
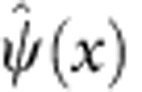
. For this purpose, we use the fact that 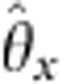 commutes for different positions and employ the polar decomposition to construct an effective field operator


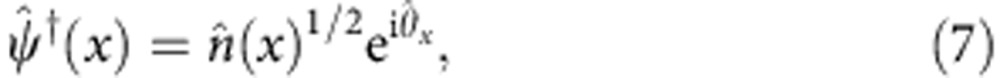


where 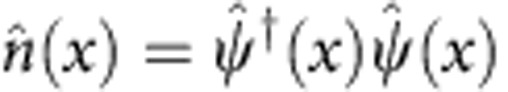 is taken to be the density of one of the two condensates. The construction ensures that these effective field operators indeed fulfil the correct commutation relations. Equation 4 follows immediately.

In the cMPS formalism, the translationally invariant correlation functions in equation 4 can be directly calculated in terms of the cMPS variational parameter matrices *R* and *Q* in the thermodynamic limit as


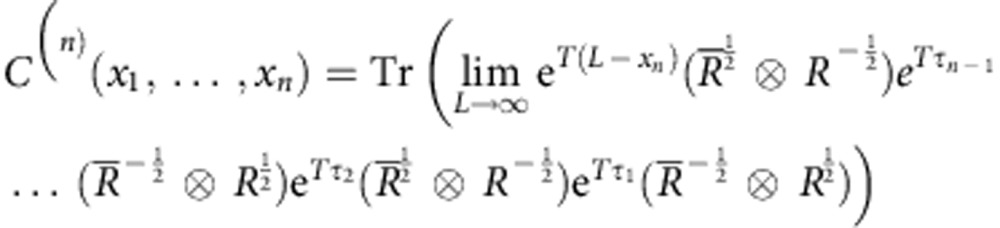


with the transfer matrix


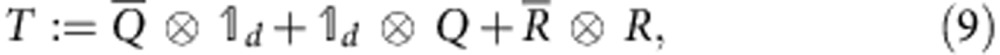


and positive distances *τ*_*j*_=*x*_*j*+1_−*x*_*j*_ for *j*=1,…,*n*−1. The overline denotes complex conjugation. This form of the correlator can be derived by the correspondences between field operators and variational matrices as described in refs 18, 19.

By writing all the matrices in the basis where the transfer matrix *T* is diagonal and performing the limit *L*→∞, the correlation function takes the form


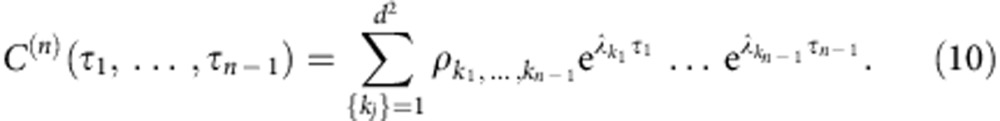


The *λ*_*k*_ are the eigenvalues of the transfer matrix *T*, also known as poles and the pre-factors, usually refered to as residues, are


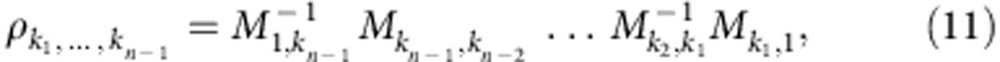


with


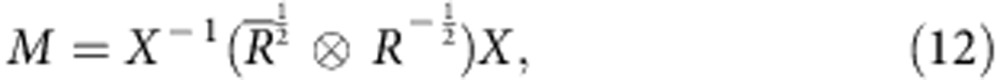


where *X* has been chosen such that *X*^−1^*TX* is diagonal^16^,^26^. For a fixed bond dimension, there are in general *d*^2^ poles and 
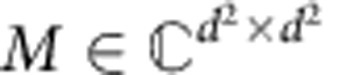
. Note that this is different from the definition in ref. 26 where the matrix *M* stems from density-like correlation functions


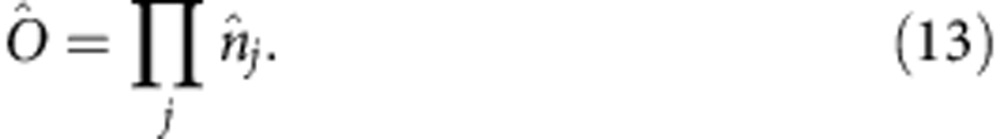


There, according to the calculus of cMPS correlation functions, the field operator term for each position corresponds to the matrix 
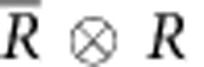
.

Note that equating two consecutive indices *k*_*j*_, *k*_*j*+1_ in the *n*-point function in equation 10 leads to a (*n*−2)-point function, as expected from equation 2. Specifically, there are many equivalent projections of a four-point function that correspond to two-point functions. However, due to imperfections (that is, deviations from translational invariance), the experimental realizations of these projections are not identical. Averaging over the projections leads an expression of the same form of a two-point correlation function from a translationally invariant cMPS as follows,


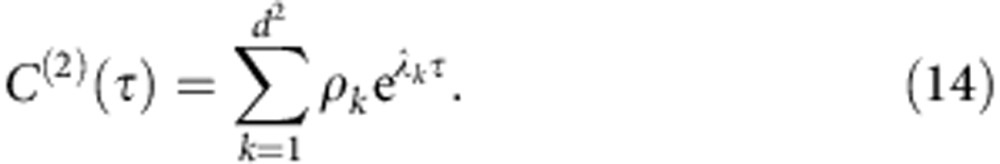


The reconstruction starts by extracting the eigenvalues *λ*_*k*_ from the averaged two-point correlation function using a least-squares fit and under the assumption of translational invariance for the modelled system. The suitable bond dimension for the data at hand can already be judged at this point, by analysing the structure of the two-point correlation function. To determine all entries of *M*, *n*-point functions with *n*>2 have to be taken into account, since for *n*=2, only the entries *M*_1,*k*_^−1^ and *M*_*k*,1_ appear, see equation 15. Since multiplying *M* with a constant and conjugating it with a diagonal matrix whose first entry is equal to one leaves all properties considered in this work invariant, we can require that *M*_1,*k*_=1 for each *k*=1,…, *d*^2^ (refs 16, 26). The remaining independent entries of the *M* matrix are fixed by included four-point correlation data. For this, we use a Nelder–Mead simplex algorithm that varies the parameters of the *M* matrix, and calculates the corresponding residues according to


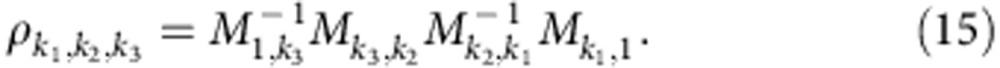


Each choice of an *M* matrix thus gives a prediction for the four-point correlators and the agreement with the experimental data is taken as the quality indicator for the algorithm. Working with a cMPS with bond dimension *d*=2 and relying on a set of 100 random initial numerical seeds proved to be sufficient for approximating the measurement data well. Taking into account the gauge and symmetry arguments^26^, the employed cMPS, with bond dimension *d*=2 in terms of *λ*_*k*_ and *M*, has 15 independent parameters in total.

As discussed in the main text, we see a significant decrease of the fit quality with hold time. There are many issues entering here. One would naturally expect that entanglement entropies after the sudden quench grow over time leading to the need for a larger bond dimension. This is presumably the case, but in our analysis, this is mostly masked by two other effects. First, the statistical error in the experiment increases substantially with the hold time, making the data for longer times considerably less reliable (Fig. 2) and also questioning our fit in terms of a pure state. What is more, the translational invariance assumption is slowly violated as the hold time increases. This is not surprising, since the light-cone-like dynamics of the trapped system give good reason to believe that trap effects need time to enter the center part of the system. As a quantitative probe to estimate how translational invariant the data are, we consider the two-point correlation function at 21 different points and calculate the variance over those different positions for variable distances. The mean of those variances gives a good indicator on how much the two-point function varies depending on the position it is evaluated at. We find for the hold times *t*=3, 7 and 23 ms deviations from translational invariance of 0.3 × 10^−2^, 5.4 × 10^−2^ and 8.3 × 10^−2^, clearly indicating that for longer hold times, our assumption of translational invariance is considerably less accurate. Given these limitations of the data set and the fact that the two-point functions averaged over different positions does not possess a rich enough structure, we feel that using a bond dimension larger than *d*=2 would be overfitting. Let us point out that this is by no means a limitation of our method as such, as reconstructions with higher bond dimension could easily be performed using matrix-pencil methods as described in ref. 26.

### Quantifying the statistical compatibility and error analysis

To quantify the error of our tomography procedure, we use the relative mean deviation with respect to the fitted (reconstructed) data,


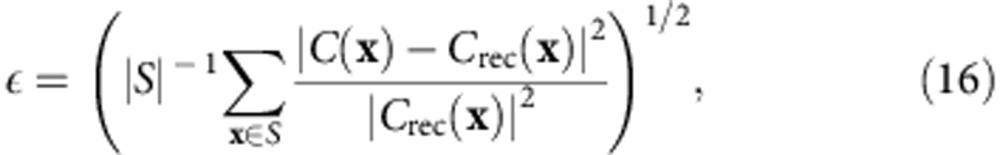


where *S* is the set of all data points **x**=(*x*_1_,…, *x*_*n*_) with *x*_1_≤*x*_2_≤⋯≤*x*_*n*_, and |*S*| denotes the number of elements in *S*. In addition, to estimate the robustness of our algorithm, we employ a bootstrapping method (see, for example, ref. 34). Namely, starting with the reconstructed four-point function from the experimental data, we add Gaussian noise with zero mean and s.d. given by the statistical uncertainties from the experiment. Subsequently, we perform our cMPS tomography procedure and reconstruct the six-point function. We repeated this procedure 100 times and computed the entry-wise relative standard deviation of the six-point functions. For the average over all entries, we obtain a deviation of 1.1% (with a maximum relative s.d. of 2.8%). This confirms that our reconstruction procedure is robust to the errors we expect in the experiment.

## Additional information

**How to cite this article**: Steffens, A. *et al.* Towards experimental quantum-field tomography with ultracold atoms. *Nat. Commun.* 6:7663 doi: 10.1038/ncomms8663 (2015).
